# Ferroptosis, a Rising Force against Renal Fibrosis

**DOI:** 10.1155/2022/7686956

**Published:** 2022-10-12

**Authors:** Yi Liu, Jingyu Wang

**Affiliations:** ^1^Department of Anesthesiology, Chengdu Second People's Hospital, Chengdu 610000, Sichuan Province, China; ^2^Renal Division, Peking University First Hospital, Peking University Institute of Nephrology, Key Laboratory of Renal Disease, Ministry of Health of China, Key Laboratory of Chronic Kidney Disease Prevention and Treatment (Peking University), Ministry of Education, Beijing, China

## Abstract

Ferroptosis is a type of programmed cell death characterized by iron overload, oxidative stress, imbalance in lipid repair, and mitochondria-specific pathological manifestations. Growing number of molecular mechanisms and signaling pathways have been found to be involved in ferroptosis progression, including iron metabolism, amino acid metabolism, lipid metabolism, and energy metabolism. It is worth noting that ferroptosis is involved in the progression of fibrotic diseases such as liver cirrhosis, cardiomyopathy, and idiopathic pulmonary fibrosis, and inhibition of ferroptosis has acquired beneficial outcomes in rodent models, while studies on ferroptosis and renal fibrosis remains limited. Recent studies have revealed that targeting ferroptosis can effectively mitigate chronic kidney injury and renal fibrosis. Moreover, myofibroblasts suffer from ferroptosis during fiber and extracellular matrix deposition in the fibrotic cascade reaction and pharmacological modulation of ferroptosis shows great therapeutic effect on renal fibrosis. Here, we summarize the latest molecular mechanisms of ferroptosis from high-quality studies and review its therapeutic potential in renal fibrosis.

## 1. Introduction

The performance of cell functions such as proliferation, differentiation, secretion, substance transport, signal transduction, and even death are essential for the normal progression and termination of organism. Cell death serves as the endpoint of cellular autoinjury to the extreme, and whether the lethal process is programmed determines the pathological or physiological significance of the pattern of death. Consequently, in a way, ferroptosis is a pathological pattern of death.

Organismal metabolism relies on various electron donors and acceptors, enzymatic/non-enzymatic reaction systems, and intact mitochondrial function to maintain redox homeostasis. The cellular responsiveness and ability to handle oxidative stress determines the degree of oxidation of proteins, lipids, DNA, etc. and thus cell fate. Oxytosis, a form of programmed cell death (PCD) directly induced by oxidative stress, is the precursor of ferroptosis [1]. Oxytosis and ferroptosis are both independent of apoptotic mechanisms and are highly consistent in terms of glutathione (GSH) depletion, lipoxygenase activation, and increased reactive oxygen species (ROS). Similarities between early studies and ferroptosis also focused on cysteine metabolism, finding that endogenous synthesis of cysteine resisted cell death [2, 3], while cysteine deficiency-induced cell death [4]. Mechanistically, cysteine is the rate-limiting substance for the synthesis of GSH, which in turn is concerned with glutathione peroxidase 4 (GPX4) abundance.

Ferroptosis was proposed in 2012, and its involvement in the pathological progression of diseases such as ischemia-reperfusion injury (IRI), stroke, inflammatory bowel disease, organ failure, and tumor progression has been widely demonstrated [5–12]. As research progressed, the following characteristics of ferroptosis were widely recognized and validated: cellular iron overload, GSH and GPX4 depletion, accumulation of lipid peroxides such as malondialdehyde and 4-hydroxynonenal and mitochondrial damage (volume reduction, outer membrane rupture, increased membrane density, and cristae reduction or disappearance) [13–15].

2019 clarified the ferroptosis regulatory mechanism—ferroptosis suppressor protein 1 (FSP1)/coenzyme Q10 (CoQ10)/NADPH axis—that is both independent and synergistic with the cystine/glutamate antiporter (System Xc-)/GSH/GPX4 axis [16, 17], hinting at the diversity of approaches to repair lipids or inhibit lipid peroxidation. The intensity of execution of lipid peroxidation is an important way to free cells from ferroptosis, and CRISPR-Cas9 and lipidomic approaches identify polyunsaturated ether phospholipids as lipid peroxidation substrates and induce ferroptosis in neurocytes and cardiomyocytes [18]. Avant-garde studies suggest that driving lipid remodeling and ether phospholipid accumulation to balance ferroptosis can also be achieved by affecting calcium homeostasis [19] and that calcium influx is also an important mechanism of oxytosis [1], suggesting that maintaining calcium homeostasis can resist oxidative stress and ferroptosis by mechanisms involving interference with lipid metabolism, mitochondrial ROS, and ferroptotic substrate production [19–21]. In fact, the molecular network of ferroptosis is expanding by leaps and bounds, which, firstly, makes its signaling mechanisms no longer to be simply categorized into GPX4-dependent and non-GPX4-dependent pathways, and secondly, makes it possible and factual to cross-link ferroptosis with a wider range of disease pathologies.

During recent years, ferroptosis has become a new favorite in the area of fibrosis prevention and treatment. However, the study of ferroptosis and renal fibrosis has been rarely reported and consequently is of great potential to be explored. Here, we review the latest studies on the regulatory mechanisms of ferroptosis and its association with renal fibrosis, hoping to enrich the prevention and treatment strategies of renal fibrosis and bring some new vigor.

## 2. The Regulatory Mechanism of Ferroptosis

### 2.1. Iron Metabolism (Figures 1 and 2)

Although the earth has abundant iron, ferrous iron is easily oxidized to ferric iron, which is hardly soluble at physiological pH, hence the bioavailability of iron is not high [22]. Iron homeostasis is related to the absorption, transport, storage, and loss of iron.

The sources of iron consist of absorption via the intestine (duodenum and upper jejunum) and destruction of senescent red blood cells [23]. The ferric iron absorbed in the intestine is reduced to ferrous iron with the help of cytochrome b and enters the cell via divalent metal transporter 1 (DMT1), then leaves the cell after intracellular shuttling and binds to the transferrin (Tf) binding site to complete retransport and distribution [24–26]. While iron destroyed by erythrocytes is mainly cleared enzymatically by macrophages and released from hemoglobin, heme oxygenase-1 (HO-1) mediates this reaction system and produces carbon monoxide and bilirubin [27, 28].

Tf travels between tissues (bone marrow, liver, skeletal muscle, etc.) to feed erythropoiesis (25-30 mg/d), and a portion of reserve iron can be mobilized in emergency situations [29]. In hemolysis and rhabdomyolysis can cause tissue iron overload due to high hemoglobin exposure [30–32]. Ferritin is the major reservoir of iron storage in the organism, and its expression level combined with Prussian blue pathological staining can reflect the degree of tissue iron overload [33]. The nuclear receptor coactivator 4 (NCOA4), which is highly enriched in autophagosomes, binds to HERC2 (E3 ubiquitin ligase) and transports ferritin to lysosomes for degradation [34, 35]. The catalytic iron released after ferritin unchaining can generate Fenton and Haber-Weiss reactions with hydrogen peroxide, leading to hydroxyl radical and peroxide production and destruction of cell membrane phospholipids, which is an important mechanism by which ferritinophagy triggers ferroptosis.

There is currently no consensus on the pros and cons of ferritinophagy and its ensuing effects. Some studies suggest that ferritinophagy makes cells susceptible to ferroptosis and plays a detrimental role. Inhibition of ferritinophagy upregulates ferritin heavy chain (FtH) and GPX4, enhancing cellular resistance to ferroptosis and rescuing fibrosis and vascular injury [36–38]. Whereas, it has also been suggested that ferritinophagy is a response to intracellular iron deficiency and contributes to iron mobilization and erythropoiesis [29, 39–41]. In fact, ferritinophagy has been detected in multiple rodent disease models, such as Parkinson's disease, sepsis, diabetic complications, and acute and chronic kidney injury [33, 42–45]. Targeting ferritinophagy offers new management strategies for iron homeostasis and anemia diseases.

Iron loss in physiological conditions includes nonspecific and nonregulatory modalities, the former including transdigestive epithelial cell shedding (bile), skin cell shedding (perspiration) and urination; the latter mainly including menstruation and bleeding. Renal dysfunction, blood transfusions and administration of iron chelators can also affect urinary iron excretion [46, 47]. Consequently, iron metabolism from the perspective of the organism is rather complex and varies significantly among individuals, which is more pronounced in populations with hemochromatosis and chronic kidney disease (CKD).

The cells are heavily dependent on the iron responsive element-iron regulatory proteins (IRE-IRPs) system in optimizing iron bioavailability. Interaction of IRE with IRPs regulates mRNAs encoding iron metabolism proteins, including DMT1, FtH, transferrin receptor 1 (TfR1), and ferroportin (FPN), thereby stabilizing iron uptake, utilization, storage, and export [48, 49]. The formation of IRE-IRPs inside the 5′UTR of mRNA complexes including FPN, FtH, and ferritin light chain (FtL) represses translation, while IRPs binding to IRE in the 3′UTR of TfR1 blocks its degradation [50]. Ingeniously, the affinity of IRE-IRPs is strong in iron-deficient cells and weak in iron-sufficient cells, and this negative feedback mode of regulation ensures proper expression of genes targeted by IRPs and thus, maintains iron homeostasis. Erastin can decrease the affinity of IRE-IRPs, leading to ferroptotic cell death [51]. Nuclear factor E2-related factor 2 (Nrf2) can also optimize iron bioavailability and regulate ferroptosis by initiating gene transcription of iron metabolism-related proteins (e.g., HO-1, GPX4, ferritin) and antioxidant response element (ARE) proteins through nuclear translocation in combination with small Maf proteins [52, 53]. Certainly, other factors of the ferroptotic system, such as NADPH, GSH, and some auxiliary proteins that affect the redox status are regulated to a greater or lesser extent by Nrf2 [54, 55].

Systemic iron homeostasis is dominated by hepcidin. Hepcidin was synthesized and secreted by the liver as an antimicrobial peptide rich in disulfide bonds, encoded by the HAMP, and its expression was affected by iron homeostasis, hypoxia, and inflammation. Hepcidin binds FPN on the cell surface to internalize and degrade it, thereby blocking iron efflux [56]. In SD rats with subarachnoid hemorrhage, administration of heparin, a hepcidin inhibitor, upregulated FPN, reduced iron deposition, and restored GPX4 activity, thereby suppressing ferroptosis and preventing early cerebral injury [57]. Therefore, manipulation of the downstream of hepcidin execution, FPN, could also achieve effects on ferroptosis. FPN knockdown exacerbated erastin-induced ferroptosis in neuroblastoma cells [58], while deferoxamine (DFO) upregulated FPN to inhibit renal ferroptosis in CKD rats [33]. Although the mode of hepcidin domination of FPN is not complicated, how to stabilize hepcidin under specific pathological conditions remains to be explored in depth.

Finally, of great importance is the fact that the majority of studies are based on extreme conditions such as iron deficiency or iron overload, while the effects of subtle changes in iron status on the cell/organism are rarely reported. Moreover, understanding the order of iron processing by tissues can indicate the susceptibility of tissues to ferroptosis. These questions remain to be addressed.

### 2.2. System Xc-/GSH/GPX4 (Figure 3)

System Xc- is composed of a light chain subunit, solute carrier family 7 member 11 (SLC7A11), and a heavy chain subunit and solute carrier family 3 member 2 (SLC3A2), which are responsible for the transport of glutamate and cystine (Cys) [59]. The intracellularly ingested Cys is converted to cysteine by enzymatic reactions, followed by the final synthesis of GSH by the action of cysteine-glutamate ligase and glutathione synthase [60, 61]. The synthesized GSH can be employed as a substrate for the synthesis of GPX4 and can also bind peroxides and free radicals and be converted to oxidized GSH (GSSG) by the action of glutathione transferase, which is important for the activation of sulfhydrylases, antioxidant injury, and detoxification.

GPX4 possesses unique biological effects not found in other members of the GPXs family, as it can reduce lipid hydroperoxides to lipid alcohols and thereby repair lipids, which is directly responsible for its antiferroptotic properties. Accordingly, stable expression of GPX4 is necessary for functional maintenance; GPX4 knockout mice suffer from ferroptosis and acute kidney injury, resulting in significantly shorter survival times [62, 63]. However, GSH depletion is not required for GPX4 inactivation, like RAS-selective lethal 3 (RSL3) and ferroptosis inducing 56 can directly inhibit GPX4 [64].

A recently reemerged feature is that GPX4 is a selenoprotein and that isopentenylpyrophosphate (IPP), a product of the mevalonate (MVA) pathway, is an important pathway for the synthesis of the active center of GPX4, selenocysteine [65]. In addition, selenium synergistically activates the transcription factors (e.g., TfAP2c, Sp1) to mediate GPX4 activity, protect neurons, and improve stroke behavior [66]. Reportedly, there is crosstalk between the MVA pathway and selenoprotein coding, and long-term medication with MVA pathway inhibitors (e.g., statins) makes selenoprotein translation problematic [67]. Therefore, it is not difficult to explain the appearance of some phenotypes associated with lack of selenoprotein synthesis, such as myopathy and hepatotoxicity, in animal models of long-term statin treatment [68, 69]. Whether the use of ferroptosis inhibitors can avoid the adverse effects of long-term administration of MVA pathway inhibitors is not known, and this may be a new approach worth trying.

### 2.3. FSP1/CoQ10/NAD(P) H (Figure 3)

In fact, the origin of the FSP1 name is not the same as that of many of the more arbitrarily named compounds. Today's FSP1 is the previously known apoptosis-inducing factor mitochondrial associated 2 (Aifm2), a flavoprotein homologous to the apoptosis-inducing factor. In November 2019, Doll et al. identified Aifm2 as a potent antiferroptotic factor, which was subsequently renamed FSP1, because Aifm2 neither localizes in mitochondria nor induces apoptosis [16, 17]. The myristoylation of FSP1 contributes to the recruitment of FSP1 to the lipid bilayer, exerts oxidoreductase activity, and prevents lipid peroxide propagation to inhibit ferroptosis, which is the core mechanism of the anti-ferroptotic effect of FSP1 [16, 17].

The fully oxidized state, ubiquinone (CoQ), and the fully reduced state, ubiquinone (CoQH2), are the two statuses of CoQ10. CoQH2 is a lipophilic antioxidant that traps lipid peroxides. FSP1 inhibits lipid peroxidation by catalyzing the reduction of CoQ to CoQH2 through NADPH and NADH oxidation (NADPH predominant). Under specific conditions such as cold and *β*-receptor stimulation, FSP1 also activates NADH oxidoreductase activity, oxidizes NADH, increases NAD^+^ levels, promotes mitochondrial respiratory chain electron transmission, stimulates thermogenesis and glycolysis rates to maximize glucose oxidation, and provides fuel for the electron transport chain [70].

Recently, FSP1 has been extensively studied, especially in the field of cancer, and the FSP1 overexpressed cells exhibit cytoprotective effects in the response to RSL3 [16]. Given the low expression of GPX4 in some tumors, the induction of tumor cell death by affecting FSP1 has attracted attention, and the development of specific agonists or inhibitors of FSP1 will provide alternative opportunities for tumor therapy.

### 2.4. AMP-Activated Protein Kinase (AMPK) (Figure 3)

AMPK is an evolutionarily highly conserved serine/threonine protein kinase with important roles in the regulation of organismal metabolism. When AMPK is activated by stress, it phosphorylates downstream substrates, shutting down the synthetic pathway that consumes ATP and opening the catabolic pathway that generates ATP, and this “loop” like regulation restores the cellular energy balance. Therefore, AMPK is considered as energy sensor in eukaryotic cells [71]. There has been a proliferation of studies dedicated to AMPK over the years and it has received attention in ferroptosis.

Generally, energy stress can deplete ATP and thus drive cell death. Surprisingly, energy stress-mediated activation of AMPK mitigates IRI-mediated renal injury by inhibiting acetyl-CoA carboxylase (ACC), which in turn decreases polyunsaturated fatty acids production and thereby inhibits ferroptosis [72]. AMPK can also be activated by its upstream kinase liver kinase B1 (LKB1), suppressing lipid peroxide accumulation and ferroptosis by inhibiting phosphorylation of ACC1 and substrates required for lipid synthesis [73]. In fact, AMPK activation has been found to inhibit ferroptosis in only a few research contexts (e.g., energy stress), which requires substantial research to validate.

AMPK can also promote ferroptosis. In human cancer cell lines, AMPK mediates phosphorylation (S90/93/96 sites) of BECN1 (key regulator of macroautophagy/autophagy) and facilitates the formation of the BECN1-SLC7A11 complex to inhibit the functional activity of System Xc- [74]. Moreover, AMPK regulates Nrf2 degradation and ARE transcription, downregulates stearoyl-coenzyme A desaturase-1 and synergizes with acyl-CoA synthetase long-chain family member 4 (ACSL4) to make tumor cells susceptible to ferroptosis [75–77]. The above studies indicate that the effect of AMPK on ferroptosis is not directly achieved but is largely due to the crosstalk of key ferroptotic factors such as ACSL4, System Xc-, and Nrf2.

So can AMPK still directly affect ferroptosis? Studies show that AMPK/mechanistic target of rapamycin (mTOR) activation triggers iron overload, while AMPK/mTOR knockdown attenuates ferritinophagy and inhibits ferroptotic cell death [78–80], suggesting that AMPK regulates ferritin degradation in an autophagy-dependent manner. Mechanistically, this is associated with the release of damage-associated molecular pattern molecules and subsequent binding to receptors being influenced [81].

In conclusion, it is not contradictory that the role of AMPK in ferroptosis is dual. On the one hand, it is because of the ability of AMPK to cross-link multiple metabolisms (e.g., glucose metabolism, lipid metabolism, protein metabolism, and energy metabolism); on the other hand, AMPK activity and phosphorylation modifications are influenced by differences in cellular metabolism and energy status, which are more pronounced between paracancerous and tumor tissues. Due to the inherent and acquired resistance of tumor cells to apoptosis, inducing carcinoma cell ferroptosis is instead highly promising, and AMPK may be an effective focus point in this direction.

### 2.5. P53 (Figure 3)

P53, a transcription factor with tumor suppressive effects, regulates tumor suppression mainly through transcriptional regulation and interaction with proteins on cell cycle arrest, senescence, metabolic activities, apoptosis, and other cellular responses. In fact, p53 is mutated in the majority of human carcinomas [82].

In 2015, Jiang et al. identified SLC7A11 as a direct target of P53 by microarray screening, which correlated with the inhibitory effect of mutants within the N-terminal structural domain of P53 on SLC7A11, and conversely, SLC7A11 overexpression abrogated ferroptosis induced by the combination of p53 activation and ROS induction [83, 84]. As a consequence, p53 has been extensively reported in ferroptosis. Subsequent studies found that p53 directly activates the target gene, namely spermidine/spermine N(1)-acetyltransferase-1 (SAT1), leading to upregulation of arachidonic acid 15-lipoxygenase (ALOX15) and synergizing with ROS to cause lipid peroxidation and ferroptosis [85]. However, the exact mechanism of regulation of ALOX15 by SAT1 is unclear, which may be related to affecting levels of polyamines.

P53 also inhibits ferroptosis, which is mechanistically related to p53's action on TP53-induced glycolysis and apoptosis regulator (TIGAR) and glutaminase 2 and inhibition of ROS production [86–90]. Interestingly, late p53 activation due to SLC7A11 inhibition presents elevated cellular ROS [84], indicating that the duration of stress may be responsible for the bidirectional effect of p53 on ROS. Moreover, p53 suppresses ferroptosis by impacting the localization of dipeptidyl peptidase-4 (DDP4) and the activity of cyclin-dependent kinase inhibitor 1a (CDKN1A) [91, 92].

As a vital take of the ferroptotic system, the bidirectional regulation of ferroptosis and cell survival by p53 relies on a sophisticated microenvironment [93]. Although much tortuous work has been spent on exploring the tumor suppressive activity of p53, it has to be acknowledged that the way in which p53 interacts with upstream and downstream targets in the context of tumors is not fully clarified. Finally, the critical activity of p53 in tumor suppression remains to be explored.

### 2.6. Nuclear Protein 1/Lipocalin-2 (NUPR1/LCN2) (Figure 3)

NUPR1 is an intrinsically disordered protein of 8 kDa molecular weight, also called P8, which is lowly expressed under physiological conditions and is transcriptionally activated by stress and the tumor microenvironment [94, 95].

NUPR1 can specifically inhibit ferroptosis. NUPR1 knockoff cells have reduced viability, elevated catalytic iron levels, and increased oxidative damage and lipid peroxidation compared to wild type, which are inhibited by ferrostatin-1 (Fer-1), liproxstatin-1 (Lip-1), and DFO, whereas, apoptosis inhibitors and necroptosis inhibitors do not alter these events [96]. And consumption of the NUPR1 target gene LCN2 also promotes the ferroptotic phenotype [96]. Concordantly, knockoff/knockdown of NUPR1 in cell lines initiated lipid detoxification gene transcription [97], reduced ROS and lipid peroxidation, and did not affect Nrf2 nuclear translocation [98]. Importantly, Nrf2 knockdown also did not affect lipid detoxification gene and NUPR1 expression [98]. The above evidence suggests that NUPR1 is an antioxidant factor independent of Nrf2 and more advantageous than Nrf2 in antilipotoxicity. However, the specific effect of the NUPR1/LCN2 pathway on ferroptosis has not been extensively validated beyond carcinomas and this remains to be resolved.

## 3. Overview of Renal Fibrosis

Renal fibrosis implies the erosion of the renal parenchyma and the accumulation of scarring, and almost all chronic and progressive kidney diseases experience this pathological pathway, which is manifested microscopically by glomerulosclerosis, tubular injury, interstitial fibrosis, and capillary rarefaction. During renal injury and chronic inflammation, increased secretion/expression of relevant enzymes and inflammatory mediators, and cytokines enable fibroblasts to differentiate towards myofibroblasts, the main responsible cells for renal fibrosis, and myofibroblast proliferation enables overexpression of *α*-smooth muscle actin (*α*-SMA), collagen (COL), fibronectin, and matrix metalloproteinases (MMP) [99–102].

The severity and duration of injury/stress exposure determines the possibility of fibrotic matrix being absorbed during the repair course. When factors of renal injury persist, fibrosis progresses unchecked, and progressive deposition of extracellular matrix (ECM) causes destruction of parenchymal cell structure, reduces blood availability, and disrupts organ function. Indeed, long-term fibrosis weakens the kidney's repair capacity and ultimately causes renal failure. Although some studies consider renal fibrosis to be a pathological response secondary to renal insufficiency rather than an intrinsic factor mediating the progression of renal disease, most studies suggest that fibrosis remains a critical event in renal dysfunction and structural deterioration [100, 103].

What has to be acknowledged is that the optimal time for intervention in renal fibrosis appears to be difficult to pinpoint. When patients with CKD are hospitalized for some symptoms, kidney biopsies in a significant number of patients show severe interstitial fibrosis. With the exception of a minority of patients with mild symptoms and a tendency to self-heal from kidney disease, most patients are administered agents immediately to improve renal microcirculation after their diagnosis is clear, and although this effectively avoids the deterioration of renal function in the patient population, it somehow makes the organism less capable of endogenous antifibrosis. Therefore, the timing of antifibrotic interventions without sacrificing endogenous intrinsic resistance is also important.

Previous accumulated data indicate that the progression of renal fibrosis implicates various PCDs such as apoptosis, autophagy, and necrosis [104–106]. However, the relationship between ferroptosis and renal fibrosis is not entirely clarified, and it is necessary to discuss the role of ferroptosis in renal fibrosis.

### 3.1. Renal Fibrosis and Ferroptosis (Figure 4)

Although ferroptosis has been proposed for 10 years, its exploration with renal fibrosis has been extremely limited. Many of the pathophysiological mechanisms of renal fibrosis are highly consistent with fibrotic diseases such as liver cirrhosis, cardiomyopathy, and idiopathic pulmonary fibrosis, and ferroptosis has been found in all of these fibrotic diseases, suggesting that ferroptosis is a nonspecific mode of death in fibrotic diseases.

Kidney cells contain a variety of iron metabolism proteins for iron ion exchange, except for some iron ions that are absorbed back into the plasma under physiological conditions and the rest is excreted by the urine without being deposited in the kidney. However, this becomes a prerequisite for the occurrence of ferroptosis under some pathological conditions. Multiple studies have discovered ferroptotic phenotypes during chronic kidney injury from IgA nephropathy (IgAN), membranous glomerulonephritis (MN), crystalline nephropathy, and diabetic nephropathy (DN) [107–117]. In contrast to its dual-edged role in the progression of liver fibrosis [118], ferroptosis is thought to play an unfavorable role in renal fibrosis, mediating renal cell death. Therefore, ferroptosis is considered as a new strategy to inhibit renal fibrosis and salvage kidney lesions.

In a study by Zhao et al., human tubular epithelial cell (TEC) mitochondria from IgAN and MN were assayed and the mitochondrial morphology of these two groups of patients showed smaller size, ruptured outer membrane, and partial loss of mitochondrial cristae compared to the control group [119]. In addition, their immunohistochemical data demonstrated that ACSL4 was highly expressed in impaired TECs and that the expression level was positively correlated with the patient's serum creatinine and blood urea nitrogen levels and negatively correlated with estimated glomerular filtration rate [119]. However, the evaluation between ferroptosis and CKD patients needs to be validated in a larger population to elucidate its generalizability. Further, single-cell RNA sequencing of the TEC population in the mouse model of kidney injury revealed that ferroptosis genes (e.g., GPX4, ACSL4, and heat shock protein beta-1 (HSPB1)) were more predominant in damaged TECs than pyroptosis- and necroptosis-related genes [119]. In fact, ferroptotic biochemical trends was also found in kidney biopsy specimens from DN patients [109]. Collectively, above studies suggest that ferroptosis is one of the inherent forms of renal cell death in various types of CKD. Although ferroptosis has been clearly found to be involved in renal failure, CKD kidneys still retain some compensatory capacity before proceeding to end-stage renal disease (ESRD), and it is still essential to follow up renal ferroptosis in a large sample of CKD patients at different stages, which will provide a reference for the timing of interventions targeting ferroptosis.

Currently, ferroptosis has been found in unilateral ureteral obstruction (UUO) and 5/6 nephrectomy rodent model of renal fibrosis [33, 120, 121], which combined with previous kidney biopsy specimens from CKD patients suggest that renal ferroptosis is a deteriorating factor contributing to kidney fibrosis. In the study by Zhou et al., WB displayed a faster postoperative downward trend of GPX4 in IRI mice compared to UUO mice, and it was still not restored at postoperative day 28 [121], suggesting that ferroptosis occurs slowly in the context of CKD (iron deposition and lipid peroxidation take longer), and also that the altered hemodynamic rhythm serves as a worsening factor for GPX4 depletion. In a 5/6 nephrectomy-induced CKD rat model, downregulation of GPX4 and SLC7A11, and upregulation of ACSL4 and specific mitochondrial changes were observed together with renal iron accumulation, oxidative stress, and lipid peroxidation in rats at week 8 postoperatively, and residual kidney iron metabolism disorders (FPN downregulation and NCOA4 upregulation) was an important mechanism for ferroptogenesis [33]. Cisplatin and DFO target ferroptosis to affect COL I and *α*-SMA deposition thereby exacerbating or ameliorating renal fibrosis [33]. Indeed, objective modeling discrepancies between UUO and 5/6 nephrectomy make the residual kidney compensatory capacity quite different, and cell death may change at different time points, and further tracking of these changes will be necessary. Furthermore, Fer-1 and DFO decrease tubular inflammatory cell chemotaxis and thus, counteract renal injury and fibrosis by inhibiting ferroptosis [121]. Ferroptosis-specific inhibitors have also shown efficacy in rodent models of hepatic and pulmonary fibrosis [122–125]. In conclusion, the above evidence suggests that ferroptosis is involved in fibrosis progression and that inhibition of ferroptosis is beneficial for tissue remodeling and regression of renal fibrosis.

Among all the CKD types, DN was the most explored with ferroptosis. DN is a major cause of ESRD in patients. ECM accumulation, mesangial expansion, glomerular basement membrane (GBM) thickening, and renal fibrosis are the typical pathological features of DN [126, 127]. The metabolic disorders, hemodynamic abnormalities, and proteinuria in DN patients make the podocytes damaged and diminished in number. Residual podocytes compensated for hypertrophy to cover the GBM, resulting in widening and/or fusion of podocytes and increased permeability of the glomerular filtration barrier, leading to massive proteinuria and further aggravating podocyte injury [128]. Therefore, podocyte injury is often considered as a contributor and predictive marker to the pathogenesis of DN [129]. Since podocytes are nonrenewable [130], prevention of podocyte autophagy and apoptosis has long been a therapeutic strategy for DN and has undeniably high potential [128, 129, 131]. A recent study found that ferroptosis occurred in high glucose-treated podocytes and that regulation of peroxiredoxin 6 expression interfered with ferroptosis and thus ameliorated podocyte injury [110]. Above data suggest that research around cell death remains a priority for tackling DN, yet the correlation between podocyte ferroptosis and DN remains limited.

Renal tubular cells are involved more in iron metabolism compared to podocytes. Reportedly, downregulation of GPX4 and SLC7A11, upregulation of ACSL4, and ferroptosis-specific changes occur in TECs in the context of DN [109]. In addition, rosiglitazone, a potent inhibitor of ACLS4, and some herbal single-agent components can affect renal COL deposition by modulating Nrf2 to alter ferroptosis resistance [111, 117]. More importantly, SLC7A11 and GPX4 were found to be downregulated in renal biopsy sections from DN patients [109], but the association of ferroptotic progression with renal insufficiency in DN patients is unknown and needs to be further explored. As research progresses, the in-depth exploration of renal ferroptosis in DN may provide new options for the treatment of DN.

Especially important is the fact that interstitial cells also express ferroptotic markers, so that myofibroblasts are as susceptible to ferroptosis as renal parenchymal cells under certain conditions. Erastin and RSL3 promote fibroblast differentiation into myofibroblasts and induce ferroptosis; Fer-1 and Lip-1 prevents this process and thus, reverses fibrosis [120, 132]. Moreover, transforming growth factor beta1 treatment decreased GPX4 abundance in fibroblast, significantly increased *α*-SMA and COL I, and presented ferroptosis-specific mitochondrial changes, suggesting that interstitial cell ferroptosis also functions in fibrosis [132]. Concurrent focus on parenchymal and mesenchymal cell ferroptosis may better resolve poor tissue repair.

## 4. Conclusion and Perspective

In the past decade, we have witnessed an explosion of ferroptosis-related research. Like other research hotspots, the misconception of ferroptosis and its flawed detection methods have made it controversial. Besides, ferroptotic markers have been found to be involved in other PCDs; therefore, specific markers and assays for ferroptosis need further refinement. Recent literature has focused on the effects and potential of ferroptosis in renal fibrosis; but how to balance ferroptosis with proliferation and differentiation of cells in renal fibrosis still requires extensive research to elucidate. That is, the role of interstitial cell ferroptosis in renal fibrosis should be of the same interset.

## Figures and Tables

**Figure 1:**
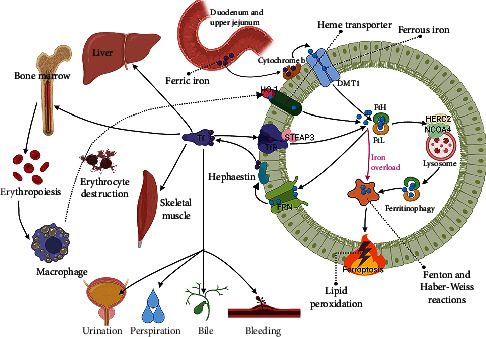
Iron metabolism and ferroptosis. Problems in any of the parts of iron absorption, transport, storage, and loss may contribute to ferroptosis. STEAP3: Six-transmembrane epithelial antigen of the prostate 3.

**Figure 2:**
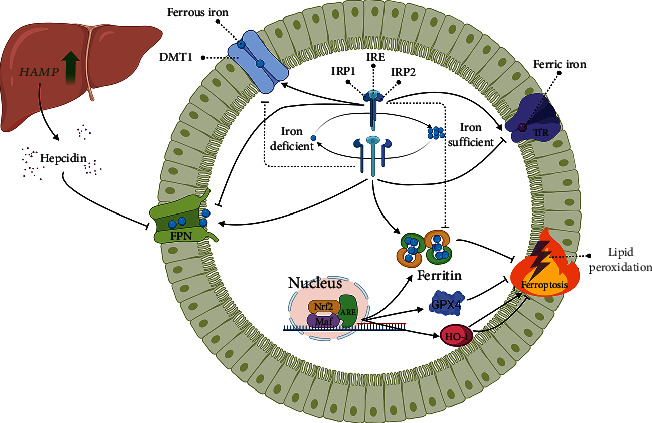
Iron regulation and ferroptosis. The common mechanisms of iron metabolism regulation are listed. The regulatory mechanisms help to correct iron metabolism disorders but are difficult to function during severe stress. Regulation of cellular iron metabolism as a critical strategy to correct ferroptosis.

**Figure 3:**
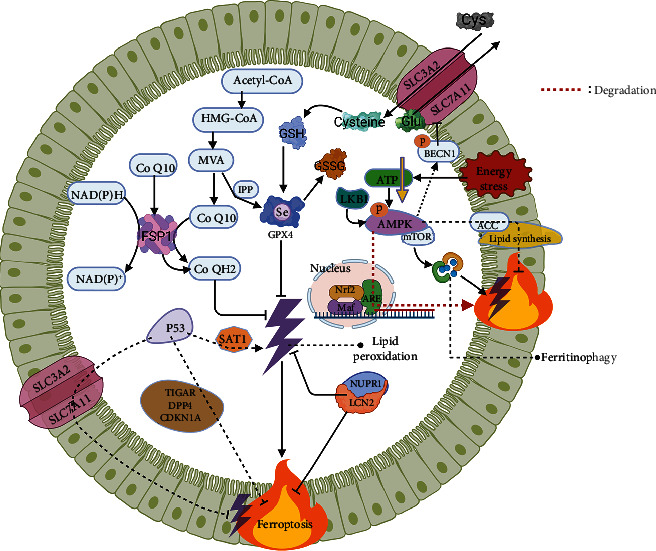
Xc-/GSH/GPX4, FSP1/CoQ10/NAD(P) H, AMPK, P53, NUPR1/LCN2, and ferroptosis. Molecules with dual ferroptotic regulatory effects (AMPK, P53) should be further dissected to elucidate their roles under specific pathological conditions. The ferroptotic network will become increasingly sophisticated and enormous.

**Figure 4:**
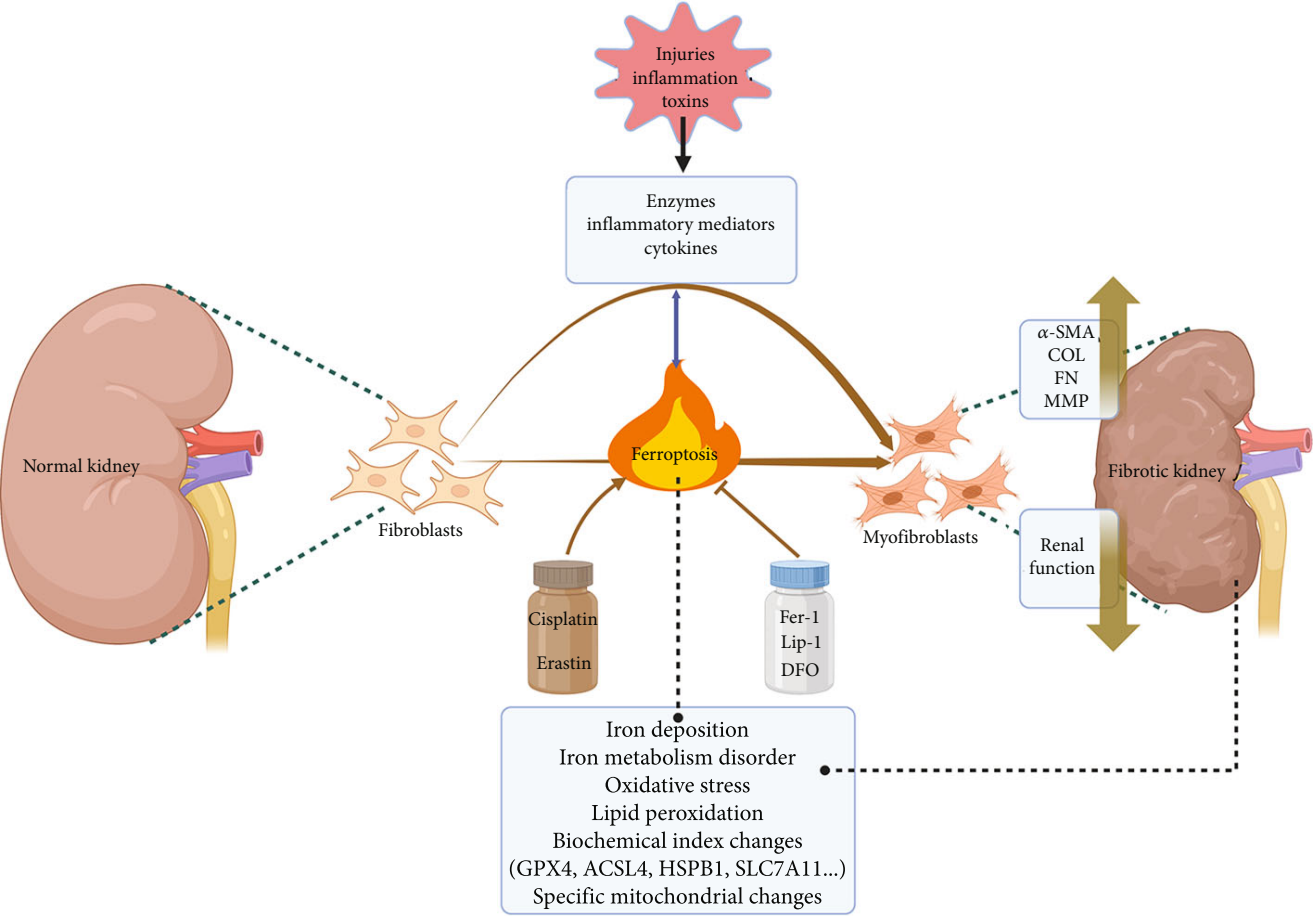
Renal fibrosis and ferroptosis. Studies have shown that ferroptosis happens during renal fibrosis and that ferroptosis also contributes significantly in the progression of renal fibrosis and promotes fibroblast differentiation. Unfavorable factors (injury, inflammation, toxins, etc.) can crosstalk with renal ferroptosis, resulting in ferroptosis-specific changes. Targeting ferroptosis for renal fibrosis and CKD has great potential.

## Data Availability

The data used to support the findings of this study are included within the paper.
